# Ziehl–Neelsen in Schistosomiasis: Much More than Staining the Shell and Species Identification

**DOI:** 10.4269/ajtmh.15-0798

**Published:** 2016-04-06

**Authors:** Soraia Vieira, Silvana Belo, Thomas Hänscheid

**Affiliations:** Instituto de Microbiologia, Instituto de Medicina Molecular, Faculdade de Medicina, Universidade de Lisboa, Lisbon, Portugal; Medical Parasitology Unit, Global Health and Tropical Medicine, Instituto de Higiene e Medicina Tropical, Universidade Nova de Lisboa, Lisbon, Portugal

The Ziehl–Neelsen (ZN) stain, also known as the acid-fast stain, has been reported to be helpful in detection and identification of *Schistosoma* eggs. 1, 2 In histological sections, *Schistosoma mansoni* egg shells appear as ZN positive and *Schistosoma haematobium* shells as ZN negative. 1, 2 The staining target of the responsible ZN component (carbolfuchsin) in the shell is unknown. Because carbolfuchsin is supposed to stain mycolic acids in the mycobacterial cell wall, 3 unidentified substances in the egg shell were proposed as target. 2 However, in histopathological examination, biopsies are exposed to concentrated ethanol and xylene during their embedding. This may alter the egg shell and allow some unusual staining reactions not seen in eggs from other sources. In fact, the ZN stain is not consistently positive in feces. 3 In addition, in some intact *S*. *mansoni* eggs, the shell was found to be only weakly ZN positive while the miracidium was found intensely positive. 3 Fuchsin is a known nucleic acid stain, 4 and it was already shown that mycobacteria with insufficiently retained carbolfuchsin may be invisible in bright-field microscopy. Yet, they can be easily detected because of a strong red fluorescence when excited with green light. 5

A smear of *S. mansoni* eggs was prestained with the nucleic acid stain 4′,6-diamidine-2′-phenylindole dihydrochloride (DAPI) and then stained with the common ZN procedure. The smear was observed (40× objective) using bright-field and fluorescent microscopy where carbolfuchsin fluoresces red (ZN-fluo) 5 and DAPI fluoresces blue (DAPI-fluo) ( Figure 1

**Figure 1.**
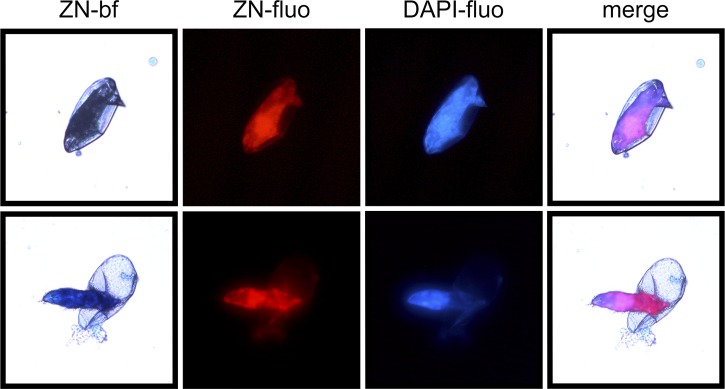
Bright-field and fluorescent image of ZN-stained *S. mansoni* eggs.

). In bright-field microscopy, the shell appears to stain very little, whereas the miracidium within the intact egg and outside the egg appears acid-fast negative, apparently only retaining the counterstain methylene blue. Contrary to this, fluorescent microscopy shows strong staining of the miracidium with carbolfuchsin (ZN-fluo) and DAPI (DAPI-fluo). The co-localization (merged) reinforces the idea that carbolfuchsin is indeed a nucleic acid stain. 4 Because acid-fast stains and low-cost light-emitting diode fluorescent microscopy are now commonly used in many regions where schistosomiasis is endemic, it may be the time to revisit the staining mechanisms of acid-fast stains 4 and investigate the use of these stains for their capacity to improve the detection of *Schistosoma* eggs.

## References

[1] Bustinduy AL, King CH, Farrer J, Hoetz PJ, Junghanss T, Kang G, Lalloo D, White NJ (2014). Schistosomiasis. Manson's Tropical Diseases.

[2] Lichtenberg F, Lindenberg M (1954). An alcohol-acid-fast substance in eggs of 
*Schistosoma mansoni*. Am J Trop Med Hyg.

[3] Richards OW (1941). The staining of acid-fast tubercle bacteria. Science.

[4] Hänscheid T, Ribeiro CM, Shapiro HM, Perlmutter NG (2007). Fluorescence microscopy for tuberculosis diagnosis. Lancet Infect Dis.

[5] Hänscheid T, Badura R, Fernandes ML, Antunes F, Cristino JM (2011). The case of the disappearing mycobacteria in Ziehl-Neelsen-stained smears. Int J Infect Dis.

